# Human *circBOULE* RNAs as potential biomarkers for sperm quality and male infertility

**DOI:** 10.7555/JBR.37.20230296

**Published:** 2024-05-29

**Authors:** Liping Cheng, He Jin, Tianheng Xiao, Xiaoyu Yang, Tingting Zhao, Eugene Yujun Xu

**Affiliations:** 1 State Key Laboratory of Reproductive Medicine and Offspring Health, Nanjing Medical University, Nanjing, Jiangsu 211166, China; 2 Center for Clinical Reproduction, the First Affiliated Hospital with Nanjing Medical University & Jiangsu Province Hospital, Nanjing, Jiangsu 210029, China; 3 Cellular Screening Center, the University of Chicago, Chicago, IL 60637, USA

**Keywords:** human *circBOULE* RNAs, sperm DNA fragmentation index, fertilization rate, cleavage rate, semen parameters, assisted reproductive technology

## Abstract

Reliable molecular biomarkers to predict fertility remain scarce. The current study investigated the potential of testis-specific *circBOULE* RNAs as biomarkers for male infertility and sperm quality. Using reverse transcription-PCR and real-time reverse transcription-PCR assays, we identified seven circular RNAs from the human *BOULE* gene in human sperm. We observed that the expression level of *circEx3-6* was significantly reduced in asthenozoospermia, while the expression levels of both *circEx2-6* and *circEx2-7*were decreased in teratozoospermia, compared with the controls. Furthermore, we demonstrated that the expression level of *circEx2-6* was negatively correlated with the sperm DNA fragmentation index, and the expression level of *circEx2-7* was correlated with both fertilization and cleavage rates in those treated with the assisted reproductive technologies. Further functional analyses in a transgenic fly model supported the roles of *circBOULE* RNAs in sperm development and human male fertility. Collectively, our findings support that sperm *circBOULE* RNAs may serve as diagnostic biomarkers for assessing sperm motility and DNA quality. Therefore, clinical application and significance of sperm *circBOULE* RNAs in the assisted reproductive technologies warrant further investigation.

## Introduction

Infertility is a pervasive global health concern affecting approximately 10%–15% of couples, with male factors contributing to half of these cases, according to the World Health Organization (WHO) ^[ 1] ^. Standard semen evaluation, a common practice in andrology laboratories, assesses the sperm fertilizing ability based on parameters such as sperm concentration, motility, and morphology. However, its diagnostic significance is limited, necessitating the identification of novel molecular biomarkers for sperm functions ^[ 2– 3] ^.

Circular RNAs (circRNAs) have emerged as promising candidates for diagnostic markers in various diseases because of their unique characteristics, including resistance to exonuclease degradation and abundance in diverse tissues. Unlike traditional linear RNAs, circRNAs, which form closed loops without free 3′ or 5′ ends, exhibit an increased stability, allowing for potential extracellular testing in biofluids and serving as novel molecular biomarkers ^[ 4] ^. Studies have reported correlations between RNA expression in sperm and male infertility conditions, such as asthenozoospermia, teratozoospermia, oligozoospermia, idiopathic infertility, and cryptorchidism ^[ 5– 12] ^. Furthermore, differences in circRNA expression have been examined among individuals with normal sperm parameters, as well as those with tumors or nerve and cerebrovascular diseases ^[ 13– 14] ^. Although the existence of circRNAs in human seminal plasma and sperm has been established, most studies have focused on broad changes in circRNA expression through sequencing, lacking specificity for clinical applications ^[ 15– 17] ^. Thus, a widely used biomarker for male sperm RNA in clinical settings remains to be investigated.

*BOULE*, a member of the human deleted in azoospermia ( *DAZ*) family, is crucial for spermatogenesis and is evolutionarily conserved from sea anemones to humans ^[ 18– 20] ^. circRNAs derived from *BOULE* locus ( *circBOULE* RNAs) are also conserved and play a role in long-term fertility preservation in *Drosophila* and mice in response to heat stress ^[ 21] ^. While previous studies reported the expression of *circBOULE* RNAs in human sperm and their correlation with asthenozoospermia ^[ 21] ^, the prevalence of these RNAs across various clinical sperm parameters of infertility remains uninvestigated. Therefore, the current study aimed to identify all *circBOULE* RNAs and determine potential correlations between their expression levels and sperm quality, as well as the assisted reproductive technology parameters and outcomes, by characterizing and analyzing their abundance in the sperm samples from reproductive clinics in Jiangsu, China.

## Materials and methods

### Human tissue samples

The current human study was approved by the Institutional Review Board of Nanjing Medical University and was performed after obtaining a written informed consent from each participant. The study subjects were recruited from Jiangsu Province Hospital (Nanjing, China). Human testis tissues were collected from a healthy middle-aged man with a normal function of spermatogenesis during the treatment of a testicle injury. The tissues were transported on dry ice immediately after dissection and were preserved in liquid nitrogen. Semen samples were obtained from men who provided a written informed consent at the Center for Clinical Reproduction, Jiangsu Province Hospital. The sperm samples were allowed to liquefy at 37 ℃ for 30 min and were assessed in volume, concentration, and motility according to the WHO reference criteria by using a computer-assisted sperm analysis technology ^[ 22] ^. Subsequently, sperm samples were purified on a 40%/80% Percoll density separation.

To ensure the quality of recovered sperm RNA, we selected only sperm samples with a cycle threshold (CT) value of the internal control *GAPDH* equal to or less than 28 for further analysis. We collected 247 semen samples from males, of which 108 samples met the criteria for sperm purity and RNA quality and were included in the *circBOULE* expression analysis. The semen analysis was performed according to the WHO guidelines (2010) ^[ 22] ^: 33 samples were included as a control group with normal semen profiles, with progressive sperm (PR) > 32%, sperm motility (SM, PR + non-progressive [NP]) > 40%, sperm concentration (SC) > 15 × 10 ^6^/mL, total sperm number (TSN) > 39 × 10 ^6^/ejaculate, normal sperm morphology rate (NSMR) > 4%, and DNA fragmentation index (DFI) < 15%; 65 samples with PR ≤ 32% or 62 samples with SM ≤ 40% were recorded as asthenozoospermia; 11 samples with SC ≤ 15 × 10 ^6^/mL or six samples with TSN ≤ 39 × 10 ^6^/ejaculate were recorded as oligozoospermia; and nine samples with NSMR ≤ 4% were recorded as teratozoospermia. The age and semen parameters of the controls and patients with PR ≤ 32%, SM ≤ 40%, SC ≤ 15 × 10 ^6^/mL, TSN ≤ 39 × 10 ^6^/ejaculate, and NSMR ≤ 4% are listed in ***Table 1***. Of these, 10 samples with DFI < 15% were categorized as low DFI samples, representing average sperm DNA quality; 16 samples with median DFI (15% ≤ DFI ≤ 30%) were categorized as having poor sperm DNA quality; and 12 samples with DFI > 30% were categorized as high DFI samples, representing very poor sperm DNA quality. The age and semen parameters of these 38 samples are listed in ***Table 2***. In summary, out of the 247 total samples, 108 sperm samples passing our quality control were selected, including 33 control samples, 65 samples of PR ≤ 40%, 62 samples of SM ≤ 32%, 11 samples of SC ≤ 15 × 10 ^6^, six samples of TSN ≤ 39 × 10 ^6^, nine samples of NSMR ≤ 4% (there is overlapping among the non-control samples). DFI analyses for 38 samples were performed.

**Table 1:** Age and semen parameters of infertile patients in comparison with the control group

Parameters	Control ( *n*=33)	PR≤32% ( *n*=65)	SM≤40% ( *n*=62)	SC≤15×10 ^6^/mL ( *n*=11)	TSN≤39×10 ^6^ /ejaculate ( *n*=6)	NSMR≤4% ( *n*=9)
Age (year)	29.7±3.8	32.1±5.3 ^*^	32.0±5.4 ^*^	30.9±3.1	29.3±5.2	32.3±7.8
SV (mL)	3.9±1.3	3.7±1.6	3.7±1.7	4.7±1.5	3.1±1.6	3.8±1.9
PR (%)	53.7±9.7	22.3±7.9 ^***^	21.9±7.9 ^***^	26.1±15.5 ^***^	21.2±13.6 ^***^	26.2±22.0 ^***^
SM (%)	69.6±10.9	29.5±10.1 ^***^	28.5±9.7 ^***^	33.4±17.4 ^***^	28.8±18.4 ^***^	35.5±27.0 ^***^
SC (10 ^6^/mL)	99.1±68.7	54.1±52.8 ^***^	53.8±53.6 ^***^	9.7±3.1 ^***^	10.7±5.3 ^**^	58.4±48.4
TSN (10 ^6^/ejaculate)	357±211	179±161 ^***^	175±161 ^***^	47±24 ^***^	28±7 ^***^	204±211
Values are expressed as mean ± standard deviation. ^*^ *P* < 0.05, ^**^ *P* < 0.01, and ^***^ *P* < 0.001 by Student's *t*-test, compared with the control group. Abbreviations: SV, semen volume; PR, progressive sperm; SM, sperm motility; SC, sperm concentration; TSN, total sperm number; NSMR, normal sperm morphology rate.						

**Table 2:** Age and semen parameters of patients based on DFI

Parameters	DFI<15% ( *n*=10)	15%≤DFI≤30% ( *n*=16)	DFI>30% ( *n*=12)
Age (year)	29.9±3.1	30.8±3.4	36.9±6.9 ^**^
SV (mL)	3.9±1.5	3.3±0.8	3.7±1.3
PR (%)	91.2±42.4	77.6±63.0	64.5±90.2 ^**^
SM (%)	43.2±21.8	30.5±11.3	23.0±9.9 ^**^
SC (10 ^6^/mL)	59.5±27.2	39.0±14.5 ^*^	31.8±12.3 ^**^
TSN (10 ^6^/ejaculate)	373±240	260±214	202±246
DFI > 30% (high), 15% ≤ DFI ≤ 30% (median), DFI < 15% (low). Values are expressed as mean and standard deviation. ^*^ *P* < 0.05 and ^**^ *P* < 0.01 by Student's *t*-test, compared with the low DFI group. Abbreviations: DFI, DNA fragmentation index; SV, semen volume; PR, progressive sperm; SM, sperm motility; SC, sperm concentration; TSN, total sperm number.			

### Isolation of sperm from human semen

A 40%/80% Percoll density gradient was used to purify the sperm in seminal plasma. The sperm were collected from the lower layer of the centrifuged tube. The hematoxylin and eosin (HE) staining of the resuspended sperm was performed. To confirm the absence of somatic cells, genomic DNA, and mitochondrial DNA, we performed reverse transcription-PCR (RT-PCR) for *CD45* (a marker for somatic cell contamination), *PRM2* (by intron-spanning exonic primers), and mitochondrial *DLOOP*, respectively, with primers indicated in ***Supplementary Table 1*** (available online), and the resulting PCR products were analyzed by gel electrophoresis.

### DFI detection

We diluted the sperm sample to 1 × 10 ^6^–2 × 10 ^6^ cells/mL, then mixed sample aliquots with the acid-detergent solution. Approximately 5000 sperm cells per sample were collected and stained with acridine orange (pH 6.0). Acridine orange emits green fluorescence when bound to normal DNA (double-stranded) chromatin, and red fluorescence when bound to impaired DNA (single-stranded). A computer-interfaced flow cytometry microscope captured the fluorescence signal. The impaired DNA/total DNA rate was calculated using the formula [red fluorescence/(red fluorescence + green fluorescence) × 100%] to quantify the extent of DNA fragmentation with the value of DFI. Visualized data were shown in scatter plots and histograms, dividing these sperm cells into the main group and outer group. Because the outer group has abnormal DNA denaturation, their percentage in the whole group represents the value of DFI.

### RNA extraction

Total RNA was extracted from the collected testes or the sperm by using Trizol reagent and purified by phenol-chloroform extraction. After the DNase treatment (Promega, Madison, WI, USA), we added 45 μL DEPC water, 10 μL 3 mol/L NaAc, and 300 μL 100% ethanol into the RNA solution for precipitation at −80 ℃ overnight. The mixture was then centrifuged at 4 ℃ the next day, and the pellet was washed with 500 μL of 70% ethanol and dried at room temperature, and the sample was dissolved with DEPC water again. The RNA concentration was measured by NanoDrop 2000c (Gene, Nanjing, Jiangsu, China).

### RT-PCR or real-time RT-PCR (RT-qPCR)

DNase-treated total RNA (500 ng) was reverse-transcribed with PrimeScript RT Master Mix according to the supplied protocol (RR036A, TaKaRa Biosystems, Beijing, China) and the manufacturer's instructions. cDNAs were amplified using specific sets of primers for RT-qPCR, as listed in ***Supplementary Table 1 ***. The RT-PCR products were separated by electrophoresis.

### Generation of human *circEx3-6* RNA transgenic flies

Transgenic flies with *circBOULE* RNAs knockout (M-introns KO), genomic DNA replacement ( *gDNA-RP*), and fly *boule* *circEx2-3* ( *fly-circ2-3*) rescue were constructed as described in a previous study ^[ 21] ^. To generate wild-type or mutant human *BOULE* *circEx3-6* transgenic flies ( *h-circEx3-6* or *mut-h-circEx3-6*), fly *boule* exon2-3 in the transgenic pGEattB vector were replaced with sequences of human *BOULE circEx3-6* RNA (with or without motif2 mutations), and the pGEattB vectors were used to inject fly embryos of attP40 containing phiC-31. Transgenic progenies were scored by the presence of the red-eye phenotype. The *h-circEx3-6* and *mut-h-circEx3-6* transgenic flies were crossed with M-intron KO to examine the effect of wild-type and mutant human *circEx3-6* RNA on the fertility of M-intron KO.

### Mating test for fly fertility

One male and three *w* ^*1118*^ virgin female flies were used. All assays were performed with at least 30 replicate vials per genotype. Briefly, newly hatched unmated males were collected and kept for two days at 25 ℃ and then mated with 2- to 3-day-old virgin females for one day at 29 ℃ to allow them to "pre-mate" (offspring from the first-day mating were not counted in the fertility test). The flies were then transferred to new vials every two days at 29 ℃ to examine the fecundity by counting adult progeny numbers at different time points.

### Statistical analysis

All data for bar and line graphs were expressed as mean ± standard deviation. All experiments were repeated at least three times unless specified otherwise. Statistically significant differences between the two groups were evaluated by a two-tailed Student's *t*-test using GraphPad Prism software 5. Statistically significant differences among the three groups were evaluated by the Kruskal-Wallisovs test using SPSS software 25. The potential correlations between the seminal parameters and the relative expression of the *circBOULE* RNAs were evaluated using the Spearman correlation test, where an | *R*| value of more than 0.5 represents a strong correlation, 0.3–0.5 represents a median correlation, 0.1–0.3 indicates a low correlation, and 0–0.1 represents no correlation. A significant correlation was defined as | *R*| > 0.3. *P* < 0.05 was considered statistically significant.

## Results

### Establishment of a robust assay for circular *BOULE* RNA detection in human semen samples

**Figure 1:**
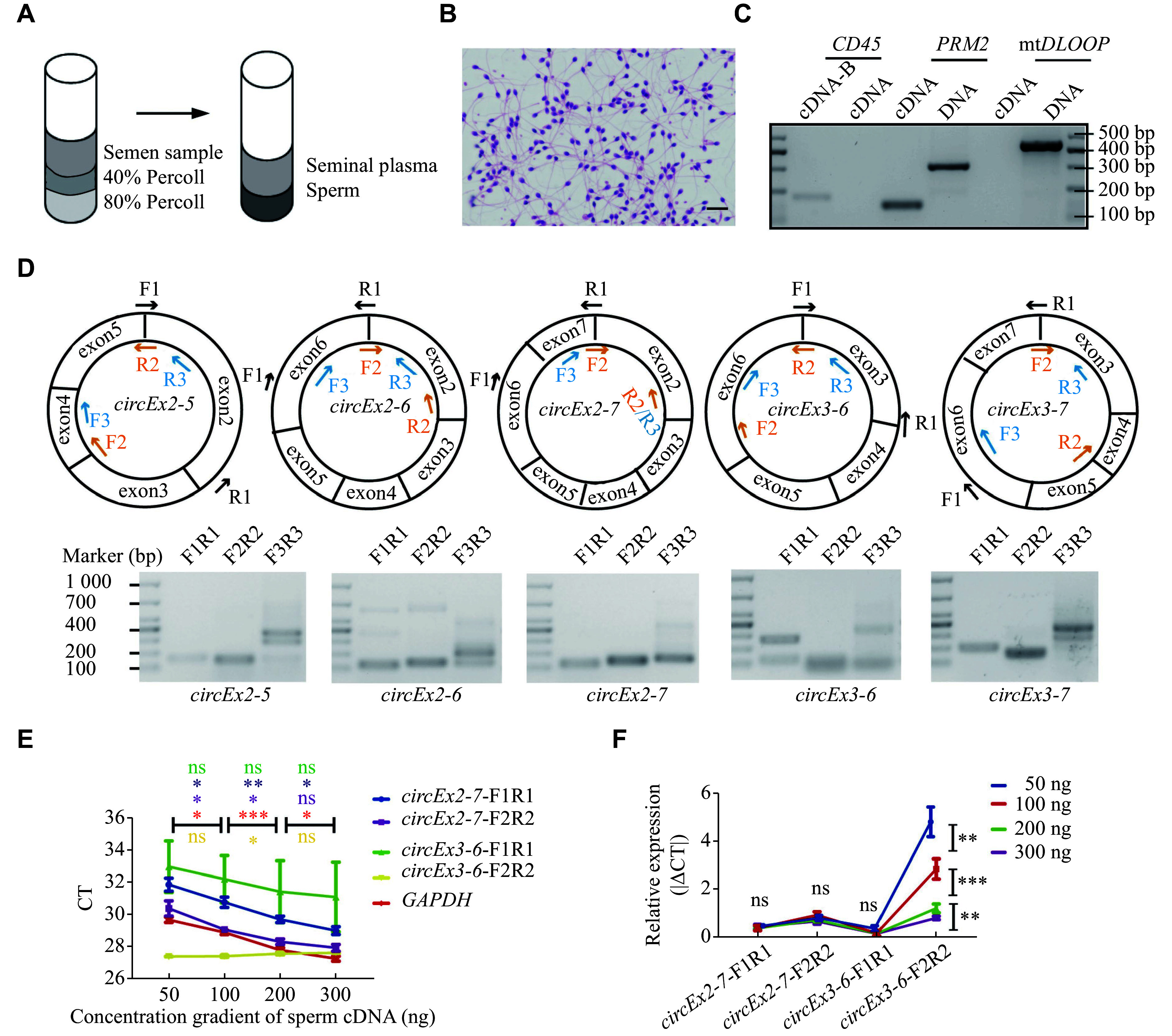
Strategies for robust detection of circular *BOULE* RNA in human sperm.

To establish an experimental method for stable detection of *circBOULE* RNAs in human sperm, we collected semen samples with normal sperm parameters from a reproductive clinic. We isolated sperm by the 80% and 40% Percoll gradient method ( ***Fig. 1A***). The purity of sperm cells and the quality of the extracted RNA were assessed by sperm smear with HE staining ( ***Fig. 1B***) and PCR analysis to detect any somatic cell gene expression or genomic or mitochondrial DNA contamination ( ***Fig. 1C***). The lack of *CD45* (a leukocyte-specific marker) PCR amplification in the sperm RNA showed that there was few, if any, leukocyte contamination in the purified sperm (99.6% ± 0.01%) ^[ 23] ^. Additionally, the absence of genomic DNA contamination was confirmed by the presence of a 148 bp *PRM2* cDNA band and the absence of a 310 bp *PRM2* genomic DNA band in the extracted sperm RNA. Mitochondrial DNA contamination was ruled out based on the absence of a 450 bp mt *DLOOP* (mitochondrial DNA marker) band ^[ 12, 24] ^. Only sperm samples that passed these quality control measures were used for the evaluation of *circBOULE* RNA expression in human sperm.

We designed three primer pairs targeting the junction and exons for *circEx2-5*, *circEx2-6*, *circEx2-7*, *circEx3-6*and *circEx3-7* RNAs, and their electrophoresis results of the PCR products are shown in ***Fig. 1D***. The results demonstrated the specificity of the primers at back-spliced junctions (forward 1 reverse 1 [F1R1], F2R2) was superior to the F3/R3 primers. Consequently, F3/R3 primers were excluded from further assays.

We further assessed the sensitivity and consistency of the RT-qPCR analysis of *circBOULE* RNAs ( *circEx2-7* and *circEx3-6* RNAs) with gradient concentrations of sperm cDNA. The CT values of *circBOULE* RNAs and *GAPDH* decreased with the increasing sperm cDNA concentration ( ***Fig. 1E***), indicating the sensitivity of the assay. When *GAPDH* was used as an internal control for PCR amplification, the relative expression levels of *circEx2-7* F1R1, *circEx2-7* F2R2, and *circEx3-6* F1R1 remained consistent for the same sperm cDNA samples despite differences in cDNA concentration ( ***Fig. 1F***). Therefore, we used the relative expression levels of circRNAs to *GAPDH* for subsequent assays of sperm *circBOULE* RNA expression. Primers like *circEx3-6* F2R2, exhibiting more variability, were excluded from further assays.

### Identification of seven *circBOULE* RNAs from the human *BOULE* locus in human sperm and testis samples

**Figure 2:**
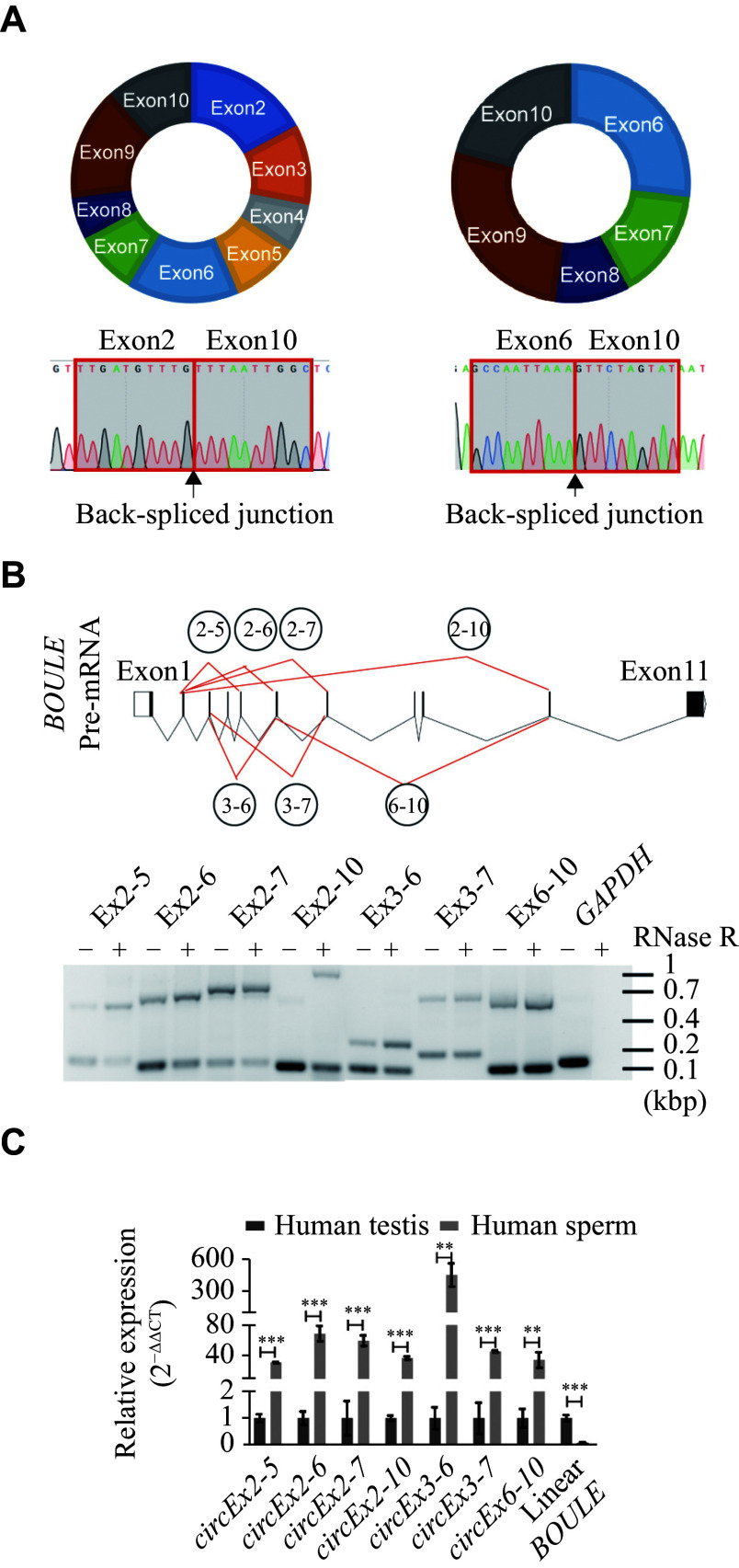
Identification of seven *circBOULE* RNAs in human sperm and testis samples.

Studies have demonstrated that circRNAs result from the back-splicing of various exons during splicing ^[ 21, 25] ^. In our previous work, we identified five *circBOULE* RNAs ( *i.e.*, *circEx2-5*, *circEx2-6*, *circEx2-7*, *circEx3-6*, and *circEx3-7*) from human sperm samples ^[ 21] ^. To detect additional circRNAs originating from the human *BOULE* locus, we designed primers spanning different exon regions. Apart from the previously identified *circBOULE* RNAs, two additional *circBOULE* RNAs, *circEx2-10* and *circEx6-10*, were identified through Sanger sequencing ( ***Fig. 2A***) and RT-PCR ( ***Fig. 2B***). The expression levels of these *circBOULE* RNAs in normal human sperm and testis samples were further analyzed using RT-qPCR. Notably, the expression level of *circEx3-6* RNA was approximately 500 times higher in the sperm than in the testis ( ***Fig. 2C***), whereas the expression levels of other *circBOULE* RNAs were only 30 to 80 times higher in the sperm than in the testes. These significant differences between the sperm and the testis may be attributed to both the expression levels of circRNAs and changes in the expression level of the internal control *GAPDH*.

### The reduced sperm *circBOULE* RNA expression level was significantly correlated with asthenozoospermia and teratozoospermia

Oligozoospermia and teratozoospermia are prevalent causes of male infertility worldwide ^[ 6– 8, 12] ^. Previous investigations indicated lower *circBOULE* RNA levels in asthenozoospermia from a single *in vitro* fertilization (IVF) clinic ^[ 21] ^, yet the broader implications of this observation remain unknown.

To investigate the potential of *circBOULE* RNAs as biomarkers for sperm quality and male infertility, we first compared the expression levels of *circEx2-5*, *circEx2-6*, *circEx2-7*, *circEx3-6*, and *circEx3-7* RNAs between the control group and each infertility group based on sperm parameters. The results showed that the expression level of *circEx3-6* RNA was significantly lower in asthenozoospermia sperm samples than in the control samples ( *n* = 33), with a reduction of approximately 54% for PR ≤ 32% ( *n* = 65) or 52% for SM ≤ 40% ( *n* = 62) ( ***Fig. 3A ***), whereas the expression levels of other *circBOULE* RNAs showed no significant changes ( ***Supplementary Fig. 1A***, available online). Additionally, the expression levels of *circEx2-5*, *circEx2-6*, *circEx2-7*, *circEx3-6*, and *circEx3-7* RNAs showed no significant differences between the oligozoospermia sperm samples (SC ≤ 15 × 10 ^6^/mL, *n* = 11; or TSN ≤ 39 × 10 ^6^/ejaculate, *n* = 6) and the controls ( *n* = 33) ( ***Supplementary Fig. 1B***, available online). However, the expression levels of *circEx2-6* and *circEx2-7* RNAs decreased by approximately 75% and 67%, respectively, in teratozoospermia sperm samples (NSMR ≤ 4%, *n* = 9), compared with the controls ( *n* = 33) ( ***Fig. 3B*** and ***3C ***). The expression levels of other *circBOULE* RNAs were also reduced in teratozoospermia sperm samples compared with the controls, although the differences did not reach a significant level ( ***Supplementary Fig. 1C***, available online).

Given that *circBOULE* RNAs are specifically expressed in the testis and sperm, understanding the correlation between their expression levels and sperm parameters is crucial for determining their potential as biomarkers for sperm quality and/or human infertility. Therefore, we analyzed correlations between *circBOULE* RNA expression levels and patients' semen parameters by Spearman correlation analysis. Although the absolute Spearman's relation value (| *R*|) of age, semen volume, PR, SM, SC, and TSN to *circBOULE* RNA was not high (all | *R*| < 0.3), the correlation between *circEx3-6* expression levels and progressive motility or sperm motility among 107 samples was statistically significant ( ***Supplementary Table 2 ***, available online). This finding is consistent with previous studies and further supports *circEx3-6* as a biomarker for asthenozoospermia ^[ 21] ^.

### Correlation between *circEx2-6* expression level and sperm DFI

Men with abnormal semen parameters often exhibit a poor sperm DNA quality and an increased sperm DNA fragmentation ^[ 26] ^. Assessing sperm DFI is crucial for evaluating sperm DNA integrity. In the current study, we analyzed the expression of *circBOULE* RNAs ( *i.e.*, *circEx2-5*, *circEx2-6*, *circEx2-7*, *circEx3-6*, and *circEx3-7*) in 38 samples from patients with different levels of DFI. *circBOULE* RNA levels showed no significant changes between patients with low DFI (DFI < 15%, *n* = 10) and medium DFI (15% ≤ DFI ≤ 30%, *n* = 16), whereas the expression level of *circEx3-6* RNA in the sperm from patients with high DFI (DFI > 30%, *n* = 12) was significantly decreased by approximately 48% ( *P* < 0.05), compared with low DFI sperm, but not observed for *circEx2-5*, *circEx2-6, circEx2-7*, and *circEx3-7* RNAs ( ***Fig. 3D*** and ***3E ***, ***Supplementary Figs. 2A*** and ***3*** [available online]). However, within the entire study population ( *n* = 38), negative correlations were observed between the expression levels of *circEx2-6* (|R| > 0.3, *P* < 0.01), *circEx3-6* (|R| > 0.3, *P* < 0.05), and *circEx2-5* RNAs (|R| > 0.3, *P* < 0.05) and DFI, whereas no such correlation was found between DFI and either *circEx2-7* or *circEx3-7* RNA ( ***Fig. 3F*** and ***3G ***, ***Supplementary Fig. 2B*** [available online]).

Additionally, among the 38 male infertility patients, those with a high DFI had a significantly lower PR and SM, compared with those with a low DFI ( *P* < 0.01) ( ***Table 2***). Lower DFI values correspond to higher sperm DNA quality. Thus, the expression levels of *circEx2-6* RNA and *circEx3-6* RNA may be significantly associated with sperm DNA integrity.

### Correlation between sperm *circBOULE* RNA expression levels and IVF embryo parameters

A high DFI may compromise sperm function and fertilization outcomes ^[ 23, 26] ^. Previous studies demonstrated that the absence of *circBoule* in mouse sperm resulted in abnormalities in pronuclear and two-cell embryo formation ^[ 21] ^. Therefore, we investigated potential correlations between *circBOULE* RNA expression levels and fertilization rates in patients undergoing the assisted reproductive technology (ART) treatment.

In the current study population, the expression level of *circEx2-7*, but not *circEx2-5*, *circEx2-6*, *circEx3-6*, and *circEx3-7*, showed a significant positive correlation with a fertilization rate (| *R*| > 0.3, *P* < 0.05, *n* = 28) ( ***Fig. 3H*** and ***Supplementary Fig. 4A*** [available online]). A positive correlation between expression level *circEx2-7* (| *R*| > 0.3, *P* = 0.038, *n* = 28) and cleavage rate was also demonstrated, but not observed for *circEx2-6*, *circEx2-5*, *circEx3-6*, and *circEx3-7* ( ***Fig. 3I*** and ***3J ***, ***Supplementary Fig. 4B*** [available online]).

It is demonstrated that sperm genome integrity is crucial not only for a successful fertilization but also for embryogenesis ^[ 27– 28] ^. However, when comparing patients with a successful gestation ( *n* = 7) and those with a failed gestation ( *n* = 9), no significant difference in DFI was observed ( *P* = 0.394). Examination of sperm circRNA expression levels in relation to fertilization and cleavage rates of the embryos revealed a strong correlation, because four out of five showed a significant correlation between circBoule expression and fertilization rate and cleavage rate ( ***Supplementary Table 3 ***, available online). Furthermore, there was no significant difference in *circBOULE* RNA expression levels between patients with a successful gestation ( *n* = 7) and those with a failed gestation ( *n* = 9) following the ART treatment ( ***Supplementary Fig. 4C***, available online). In summary, while *circEx2-7* RNA may be a potential biomarker for the fertilization rate for IVF in the reproductive clinic, larger sample sizes and multi-center studies are needed to validate our findings.

### Functional study of *circBOULE*: human *BOULE* *circEx3-6* RNA partially rescued fertility in *circboule* RNAs KO flies

**Figure 4:**
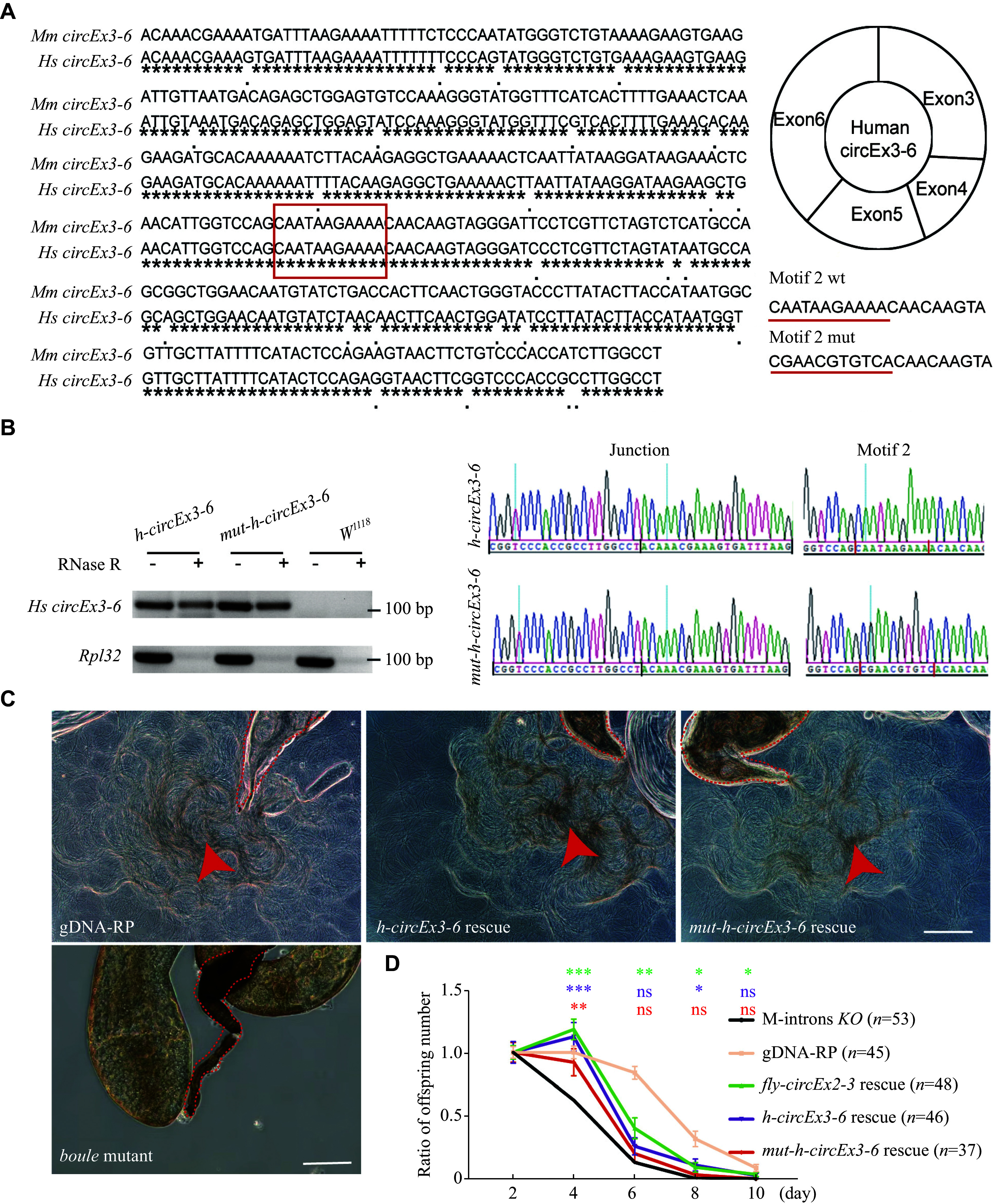
Human *circBOULEEx3-6* partially rescued the fertility defects in *circBoule* RNA deleted (M-introns KO) fly.

To elucidate the function of human *circBOULE* RNAs, we used the *Drosophila* model to express these human *circBOULE* RNAs. Sequence alignment revealed that the sequences of *circBOULEEx3-6* RNA were highly conserved between humans and mice ( ***Fig. 4A***). Interestingly, two motif sequences of human *circBOULEEx3-6* and *circBOULEEx2-7* matched that of mouse *circEx3-6*, a binding site for heat shock protein family A member 2 (HSPA2) ^[ 21] ^. Previous studies demonstrated that m- *circEx3-6* partially rescued fertility defects in M-introns KO flies, which lacked all introns between exon 2 and exon 7 of the *boule* gene ^[ 21] ^, but the functional significance of human *circBOULE* remains unknown. To investigate the functions of human *circEx3-6* RNA, several transgenic flies were constructed. The control fly strain (gDNA-RP) had the coding region of *boule* replaced with the genomic region of *boule*, and the positive control fly strain expressed fly *circEx2-3* in M-introns KO lines ( *fly-circEx2-3* rescue), as previously reported ^[ 21] ^.

The successful construction of transgenic flies expressing wild-type or mutant human *circEx3-6* RNA was confirmed by RT-PCR and sequencing ( ***Fig. 4B***). Phase-contrast images of seminal vesicles, where mature sperm are stored, equivalent to the mouse epididymis in flies, showed that both *h-circEx3-6*and *mut-h-circEx3-6* rescue flies produced a normal amount of motile sperm ( ***Fig. 4C***).

Considering that the male fecundity of M-introns KO flies decreased significantly when young adults raised under 25 ℃ were shifted to 29 ℃, we conducted a long-term fertility assay of transgenic flies at 29 ℃. The results showed a dramatic decrease in the fertility of M-introns KO flies from the 2nd to the 8th day, with no offspring observed from the 8th to the 10th day. On the 2nd–4th day, both *h-circEx3-6* and *mut-h-circEx3-6* rescue flies exhibited a higher fertility than M-introns KO flies. However, on the 6th–8th day, the fertility of *mut-h-circEx3-6* rescue flies decreased to the level of M-introns KO flies, while *h-circEx3-6* flies maintained higher fertility than M-introns KO flies. Although the fertility of *h-circEx3-6* rescue flies was reduced in comparison with that of *gDNA-RP* (control), the fertility defect of M-introns KO flies was partially rescued by human *circEx3-6* RNA ( ***Fig. 4D***).

## Discussion

Infertility is a significant global health challenge that affects both the society and individuals. Male infertility accounts for nearly a half of infertility cases, with approximately 25% of infertile men classified as having idiopathic infertility ^[ 1] ^. Understanding the molecular mechanisms underlying male infertility is challenging, but invasive tissue biopsies are often performed only in clinics for diagnosis and treatment. The relative ease of obtaining semen samples causes minimal patient discomfort, making them a valuable resource for developing human infertility biomarkers. Our previous study highlighted the conservative roles of *circBOULE* RNAs in sperm development and maturation, with the knockout of *circBOULE* RNA in mice leading to reduced rates of pronuclear and two-cell embryo formation, and demonstrated an association between the reduced expression level of *circBOULE* and asthenozoospermia ^[ 21] ^. These findings raised the possibility of using *circBOULE* RNAs as potential biomarkers for the diagnosis and treatment of human infertility. Therefore, a detailed analysis of *circBOULE* expression in human sperm from broader populations in different reproductive clinics is needed to evaluate this possibility and to investigate the correlation between *circBOULE* expression from the entire human *BOULE* locus and various sperm quality parameters.

Recently, circRNAs have attracted a significant attention because of their unique circular structure and diverse functions ^[ 4, 13, 25] ^. While our understanding of their function is still evolving, the potential diagnostic value of circRNAs has been explored in various fields, including andrology and male infertility. Several studies reported the presence of circRNAs in human semen samples and their potential correlation with male infertility ^[ 15– 17, 21] ^. In the current study, we established a robust protocol for sperm circRNA expression assay by identifying and characterizing *circBOULE* RNAs in normal human sperm. We identified seven types of *circBOULE* RNAs in human sperm, including two novel ones ( *i.e.*, *circEx2-10* and *circEx6-10* RNAs). Subsequent RT-qPCR analyses revealed alterations in the *circBOULE* RNA expression in the sperm from infertile patients, compared with the healthy controls. We found that *circEx3-6* was significantly expressed at lower levels in asthenozoospermia patients, consistent with our previous findings. This significant validation of the correlation between *circEx3-6* and asthenozoospermia in another reproductive clinic makes *circEx3-6* the first circRNA biomarker validated in more than one reproductive clinic for asthenozoospermia. Consistent with this finding, the overall *circEx3-6* expression among all the semen samples in the current study was also significantly correlated with sperm motility, further supporting *circEx3-6* as a sperm biomarker.

We further investigated the functions of human *circEx3-6* by expressing human *circEx3-6* in the *Drosophila* testis. Our previous work demonstrated that *circBoule* knockout mice exhibited a lower fertility, indicating its potential role in sperm functions. In the current study, we extended this investigation to a *Drosophila* model. Expression of human *circEx3-6* RNA in this model partially rescued fertility defects in the *circboule* knockout flies, indicating the conserved function of *circBOULE* RNA among humans, mice, and flies ^[ 21] ^and supporting the functional significance of this human *circBOULE* RNA in addition to its potential as a biomarker. Besides *circEx3-6*, we also found that the expression of sperm *circEx2-6* and *circEx2-7* was significantly lower in teratozoospermia patients but not in oligozoospermia patients.

An intriguing observation was the significant negative correlation between DFI and *circBOULE* RNA expression. DFI serves as an indicator of damaged sperm DNA fragments associated with various aspects of sperm function and embryogenesis ^[ 29– 32] ^. Additionally, significant correlations between age, conventional semen parameters, and *circBOULE* RNA were noted. These findings further support the potential of *circBOULE* RNAs as prognostic biomarkers for sperm quality and function.

However, no significant changes in *circBOULE* RNA expression levels were observed between successful and unsuccessful gestation outcomes in patients undergoing the ART treatment. The complex nature of infertility, a limited number of patients, and a lack of detailed medical history may contribute to these inconclusive results. Future investigations are warranted to explore the association between *circBOULE* RNA expression and ART outcomes.

Our previous work showed that the *circBoule* KO (intron 2 KO) mice under heat stress had a lower fertilization rate than the control, and that *circBoule* interacted with conserved heat shock proteins ( *e.g.*, HSPA2) in both fruit flies and mice to promote sperm heat shock protein degradation in the presence of heat stress ^[ 21] ^. The findings of the current study on the significantly reduced expression of human *circEx3-6* in asthenozoospermia and the strong correlation between *circEx3-6* expression and sperm motility further support the role of circular *BOULE* RNA in human sperm. The partial rescue of the fly *circBOULE* knockout phenotype by human *circEx3-6* demonstrated a functional role of human *circBOULE* RNA. Given that an aberrant HSPA2 level was significantly associated with asthenozoospermia, our data support the hypothesis that human *circBOULE* exerts its sperm function by regulating sperm HSPA2 levels, similar to its counterpart in flies and mice ^[ 21] ^. Future characterization of the *circBOULE*-HSPA2 complex is needed to elucidate the biochemical mechanism of the protective function of *circBOULE*, and to help distinguish the causal and correlative relationships between the observed *circEx3-6* expression and sperm motility. Furthermore, the specific functions of individual *circBOULE* RNAs, such as *circEx2-6* and *circEx2-7*, also require further investigation.

From this relatively large pool of human sperm samples, we identified *circBOULE* RNAs as potential biomarkers for sperm quality and fertilization in the reproductive clinics. However, we were only able to use 108 samples, because other samples either did not pass our quality control for sperm purity (33 cases), contained incomplete clinical information (18 cases), or failed in qPCR tests (15 cases). There were an additional 73 samples that were not used because of a poor amplification of the internal control *GAPDH*, considering a relatively low expression level and a high CT value in the qPCR of *circBOULE* in the sperm. Future use of a testis-specific internal control, such as *GAPDH* ( *GAPDHP72*) or *PRM2*, which are expressed at higher levels in the sperm than the ubiquitous *GAPDH* used in the current study, may improve the sensitivity of our assays and hence reduce the loss of samples that were otherwise discarded because of the poor amplification of internal control like *GAPDH* ^[ 5, 33] ^. Although more sensitive sperm internal controls should not change the observed correlation between *circEx3-6* expression and sperm motility based on ubiquitous *GAPDH*, they may significantly reduce the number of unusable samples, and increase the reliability of sperm RNA qPCR results and the number of other *circBOULE* species as potential biomarkers. Such an improvement will broaden the application of *circBOULE* for male infertility diagnostics.

The advantages of using *circEx3-6* as a biomarker for asthenozoospermia and sperm motility are severalfold. First, this *circBOULE* RNA has a high expression level in the purified sperm, often higher than in the testis, and therefore is relatively easy to detect with the standard qPCR methodology. Second, as a circRNA, the *circBOULE*RNA is much more stable than a linear mRNA or miRNA biomarker, reducing the impact of RNA degradation on the samples during sperm RNA preparation. Third, the extraordinary conservation of *circBOULE* RNAs makes it possible to study and understand their mechanistic role in sperm quality and fertilization using model organisms, unlike most of other human biomarkers. Such future investigations may, in turn, help improve the sensitivity of *circBOULE* expression assays as biomarkers and possibly reveal more sensitive novel biomarkers from the *circBOULE* regulatory pathway. The application of *circBOULE* biomarkers may complement the existing sperm parameter analysis through a computer-assisted sperm analysis and sperm quality assays like DFI.

In conclusion, the current study advances our understanding of *circBOULE* RNAs in human sperm and their potential as biomarkers for male infertility. The intricate correlations among *circBOULE* RNAs, conventional semen parameters, and fertility outcomes in humans and other species highlight the critical role of *BOULE* gene expression in male fertility and its value in dissecting the complexity of male infertility. Future investigations are needed to delineate the specific functions of individual *circBOULE* RNAs and their clinical implications for the diagnosis and treatment of male infertility.

## SUPPLEMENTARY DATA

Supplementary data to this article can be found online.
