# Supplementary material for: Human circBOULE RNAs as potential biomarkers for sperm quality and male infertility
Source: J Biomed Res. 2024 May 29;38(5):473–84. doi: 10.7555/JBR.37.20230296 (PMC11461533; doi:10.7555/JBR.37.20230296)
Supplement: Supplementary file 1 — Supplementary data to this article can be found online. [file jbr-38-5-473-S1.pdf]

Available online at www.jbr-pub.org.cn

Open Access at PubMed Central

Journal of Biomedical Research, 2024 38(5): 1–6

Supplementary Data

JBR

Human *circBOULE* RNAs as potential biomarkers for sperm quality and male infertility

Liping Cheng[1], He Jin[1], Tianheng Xiao[1], Xiaoyu Yang[2,✉], Tingting Zhao[1,✉], Eugene Yujun Xu[1,3,✉]

[1]*State Key Laboratory of Reproductive Medicine and Offspring Health, Nanjing Medical University, Nanjing, Jiangsu 211166, China;*
[2]*Center for Clinical Reproduction, the First Affiliated Hospital with Nanjing Medical University & Jiangsu Province Hospital, Nanjing, Jiangsu 210029, China;*
[3]*Cellular Screening Center, the University of Chicago, Chicago, IL 60637, USA.*

Supplementary Table 1 **Sequences of primers used for PCR or RT-qPCR**

Primers	Primer sequences (5′→3′)
h-*circEx2-5*-F2	ATTCCTCCTACAAAGATGCG
h-*circEx2-5*-R2	GTTTGATGTTTGGAGGGATCCC
h-*circEx2-6*-F1	AGCAGCTGGAACAATGTATC
h-*circEx2-6*-R1	GGTTTGATGTTTGAGGCCA
h-*circEx2-7*-F1	TCCAGAGGTAACTTCGGTC
h-*circEx2-7*-R1	GGTTTGATGTTTGCTGGTA
h-*circEx3-6*-F1	CCGCCTTGGCCTACAAAC
h-*circEx3-6*-R1	AGTGACGAAACCATACCCTTTG
h-*circEx3-7*-F1	GCAGCTGGAACAATGTATC
h-*circEx3-7*-R1	CACTTTCGTTTGTCTGGTA
h-*Ex2-10*-F	GCAGCCTGAGCCAATTAAACAAAC
h-*Ex2-10*-R	GGGGCACTTGTTGGGTTATTC
h-*Ex6-10*-F	GCCTGAGCCAATTAAAGTTCTAG
h-*Ex6-10*-R	CCGAAGTTACCTCTGGAGTATG
h-*CD45*-F	CCTTGAACCCGAACATGAGT
h-*CD45*-R	ATCTTTGAGGGGGATTCCAG
h-*DLOOP*-F	CACCATTAGCACCCAAAGCT
h-*DLOOP*-R	TGATTTCACGGAGGATGGTG
h-*PRM2*-F	TATAGGCGCAGACACTGC
h-*PRM2*-R	GCCTTCTGCATGTTCTCT
h-*GAPDH*-F	ATAAATTGAGCCCGCAGCCT
h-*GAPDH*-R	GCGCCCAATACGACCAAATC

✉Corresponding authors: Tingting Zhao, State Key Laboratory of Reproductive Medicine and Offspring Health, Nanjing Medical University, 101 Longmian Avenue, Jiangning District, Nanjing, Jiangsu 211166, China. E-mail: zhaotiti@njmu.edu.cn; Xiaoyu Yang, Center for Clinical Reproduction, the First Affiliated Hospital with Nanjing Medical University & Jiangsu Province Hospital, 16 Yongqing Street Nanjing, Jiangsu 210029, China. E-mail: yxy1921@163.com; Eugene Yujun Xu, Cellular Screening Center, Biological Science Division, the University of Chicago, 900 E 57th Street, Chicago, IL 60637, USA. E-mail: yxu@uchicago.edu.

Received: 04 December 2023; Revised: 29 April 2024; Accepted: 08 May 2024; Published online: 29 May 2024

CLC number: R321.1, Document code: A

The authors reported no conflict of interests.

This is an open access article under the Creative Commons Attribution (CC BY 4.0) license, which permits others to distribute, remix, adapt and build upon this work, for commercial use, provided the original work is properly cited.

© 2024 by Journal of Biomedical Research.

https://doi.org/10.7555/JBR.37.20230296

Supplementary Table 2 **Correlations of age, semen parameters and DFI with *circBOULE* RNA expression in the entire cohort of patients**

Variables	Parameters	*circEx2-5*	*circEx2-6*	*circEx2-7*	*circEx3-6*	*circEx3-7*	DFI
Age (year)	*R*	−0.073	−0.113	−0.053	−0.153	−0.195	0.441**
	P	0.452	0.244	0.583	0.115	0.056	0.006
	n	107	108	108	107	97	38
SV (mL)	*R*	0.174	0.176	0.167	0.165	0.009	−0.067
	P	0.072	0.069	0.084	0.09	0.931	0.689
	n	107	108	108	107	97	38
PR (%)	*R*	0.003	0.205*	0.172	0.244*	0.017	−0.391*
	P	0.972	0.033	0.074	0.011	0.869	0.015
	n	107	108	108	107	97	38
SM (%)	*R*	0.132	0.216*	0.161	0.256**	0.16	−0.464**
	P	0.177	0.025	0.097	0.008	0.117	0.003
	n	107	108	108	107	97	38
SC (10^6/mL)	*R*	0.082	0.208*	0.148	0.227*	0.12	−0.453**
	P	0.398	0.03	0.126	0.019	0.243	0.004
	n	107	108	108	107	97	38
TSN (10^6/ejaculate)	*R*	0.042	0.232*	0.196	0.269**	0.018	−0.328*
	P	0.666	0.016	0.042	0.005	0.863	0.045
	n	107	108	108	107	97	38
DFI	*R*	−0.321*	−0.395*	−0.243	−0.439**	−0.053	1
	P	0.049	0.014	0.142	0.006	0.765	—
	n	38	38	38	38	34	38

*$P < 0.05$ and **$P < 0.01$ by Spearman correlation co-efficient (*R*) test, and *n* represents the sample size
Abbreviations: SV, semen volume; PR, progressive sperm; SM, sperm motility; SC, sperm concentration; TSN, total sperm number; DFI, DNA fragmentation index.

Supplementary Table 3 **Correlations of fertilization rate and cleavage rate with *circBOULE* RNA expression**

Variables	Parameters	*circEx2-5*	*circEx2-6*	*circEx2-7*	*cirEx3-6*	*circEx3-7*
FR	*R*	0.667**	0.580*	0.606*	0.232	0.691**
	P	0.007	0.019	0.013	0.388	0.003
	n	15	16	16	16	16
CR	*R*	0.693**	0.636**	0.580*	0.229	0.620*
	P	0.004	0.008	0.019	0.394	0.01
	n	15	16	16	16	16

*$P < 0.05$ and **$P < 0.01$ by the Spearman correlation coefficient test, and *n* represents the sample size
Abbreviations: FR, fertilization rate; CR, cleavage rate.

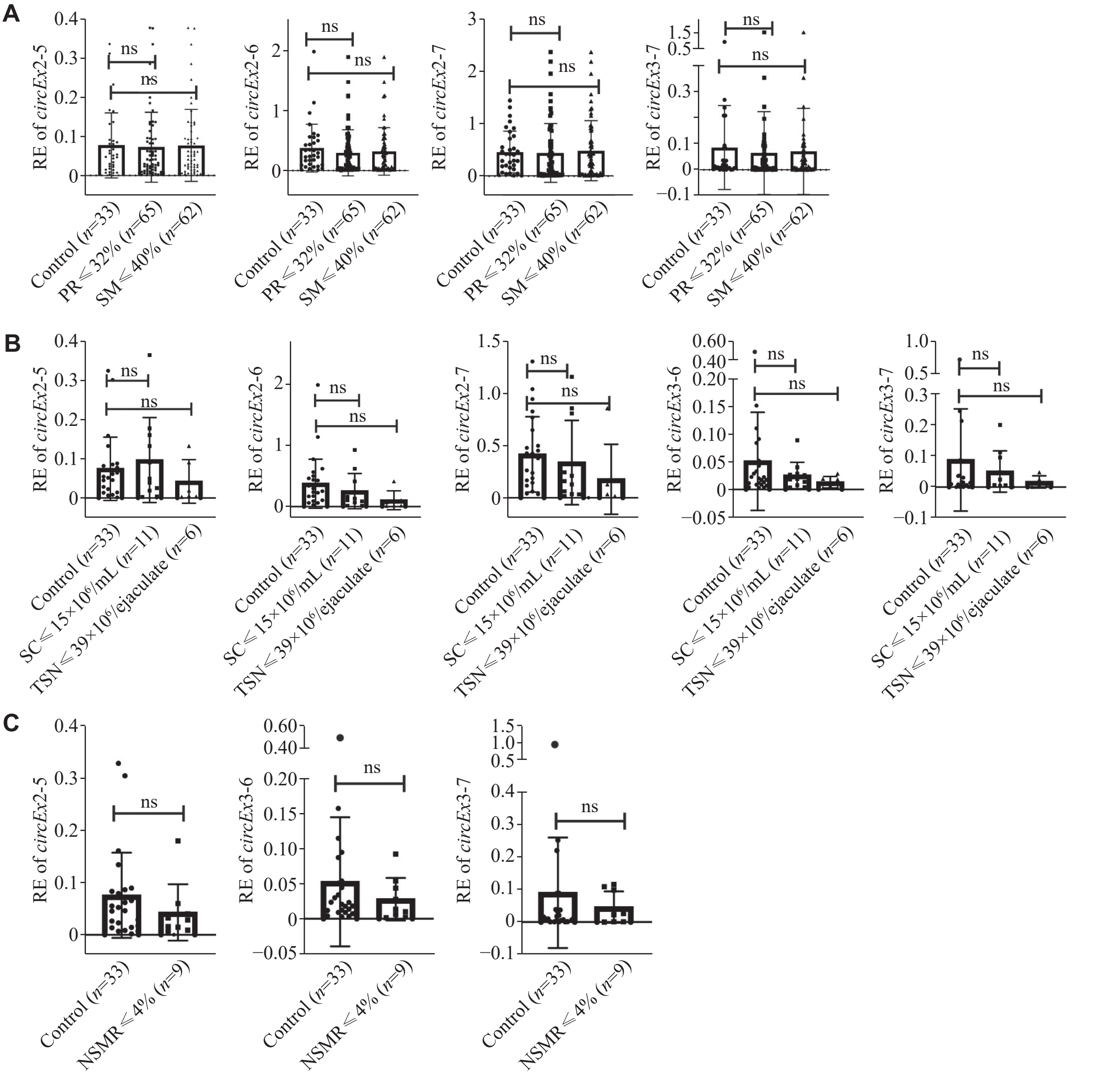

Supplementary Fig. 1 **The *circBOULE* expression levels in the sperm of reproductive clinical male patients.** A: The relative expression (RE) of *circEx2-5*, *circEx2-6*, *circEx2-7*, and *circEx2-7* in the control and asthenozoospermia groups. B: The expression of *circBOULE* RNAs in the control and oligozoospermia groups. C: The *circEx2-5*, *circEx3-6* and *circEx3-7* in teratozoospermia sperm (NSMR is less than 4%). Data are shown as mean and standard deviation. *P*-values from unpaired Student's *t*-test. Abbreviations: PR, progressive sperm; SM, sperm motility; SC, sperm concentration; TSN, total sperm number; NSMR, normal sperm morphology rate; ns, not significant.

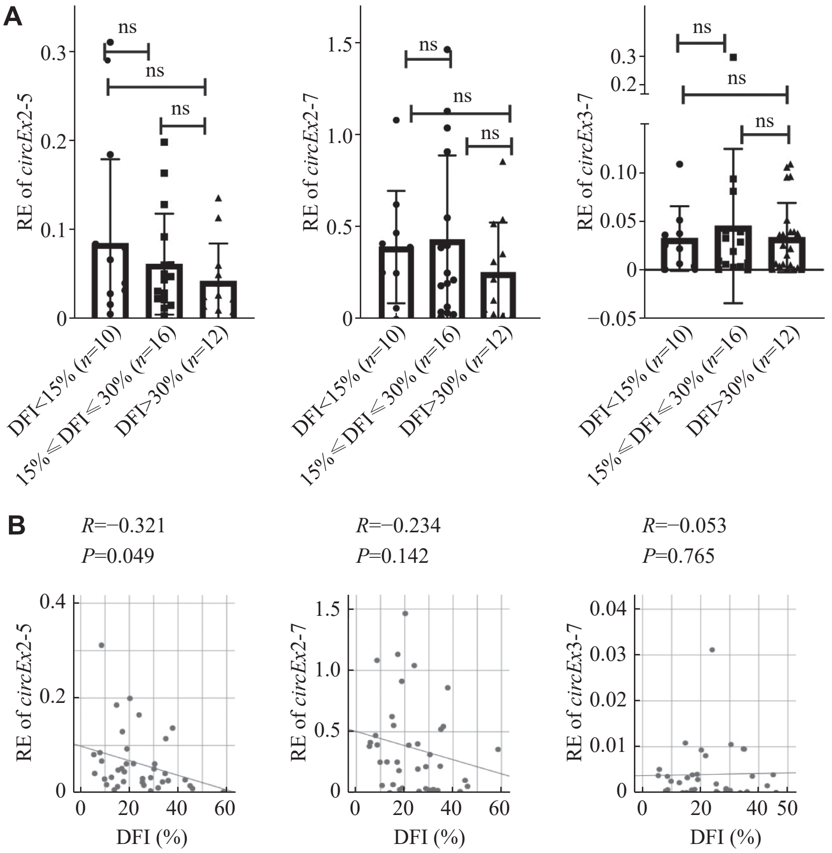

Supplementary Fig. 2 **Relative expression (RE) levels of *circBOULE* RNAs in sperm from patients with diverse DFI were assessed using real-time reverse transcription-PCR.** A: The expression levels of *circEx2-5*, *circEx2-7*and *circEx3-7* in sperm of patients with high DFI (> 30%, *n* = 12), medium DFI (15% ≤ DFI ≤ 30%, *n* =16), and low DFI (< 15%, *n* = 10). *P*-values from Kruskal-Wallis test. B: The correlations of *circEx2-5*, *circEx2-7*, and *circEx3-7* expression levels with the sperm DFIs were analyzed by Spearman correlation analysis. *R* is Spearman correlation coefficient. Data are shown as mean and standard deviation. Abbreviations: DFI, DNA fragmentation index; ns, not significant.

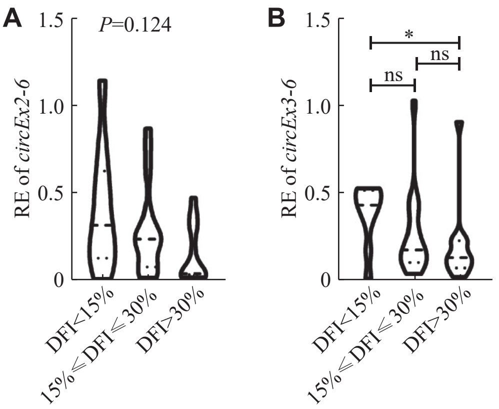

C

Parameters	1DFI<15% (n=10)	15%≤DFI≤30% (n=16)	DFI<30% (n=12)
circEx2-6	0.4±0.35	0.28±0.25	0.15±0.17
circEx3-6	0.4±0.02	0.03±0.02	0.02±0.02

Supplementary Fig. 3 **The relative expression (RE) levels of circBOULE RNAs and DFI data in the patients.** A: The violin plots showing the distribution of RE level of *circEx2-6* among the low, median, and high DFI groups, P = 0.124. P-values from Kruskal-Wallis test; dash line is for the median value, dot line is for the quartiles. B: The violin plots showing the distribution of RE level of *circEx3-6* among the low, median, and high DFI groups. P = 0.029, $P_{L\text{-}M}$ = 0.207, $P_{L\text{-}H}$ = 0.025, $P_{M\text{-}H}$ = 0.894. P-values from Kruskal-Wallis test. Dash line is for the median value, dot line is for the quartiles. C: The data of *circEx2-6* (A) and *circEx3-6* (B) expression levels among the low, median, and high DFI group.

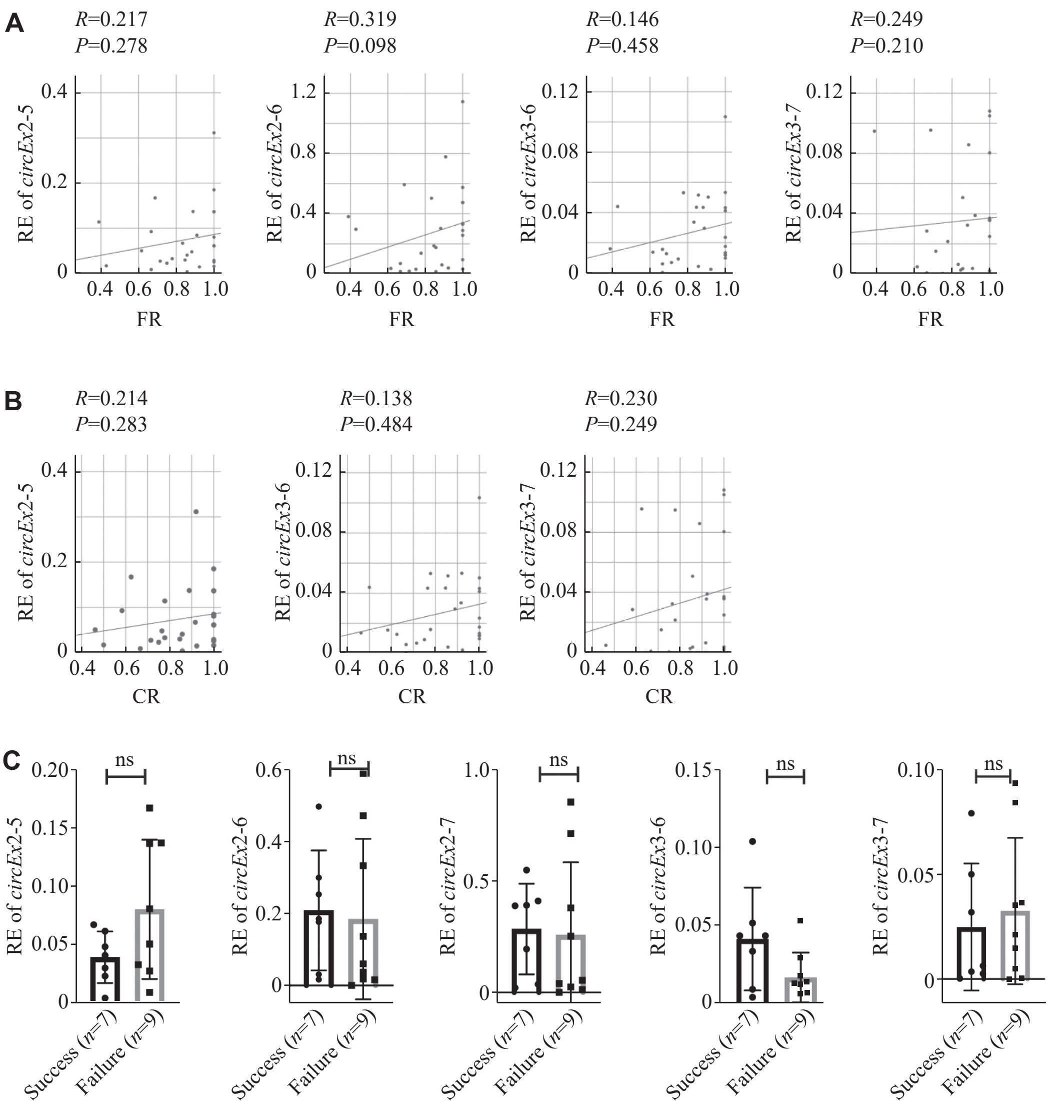

Supplementary Fig. 4 **The correlations between circBOULE and fertilization/cleavage rate (FR/CR) of patients treated with assisted reproductive technology (ART).** A: Correlations of *circEx2-5*, *circEx2-6*, *circEx3-6* and *circEx3-7* relative expression (RE) with FR of the patients treated with ART. B: Correlations of *circEx2-5*, *circEx3-6*, and *circEx3-7* with CR of the 28 patients treated with ART. *P*-values from Spearman test. C: The relative expression levels of *circBOULE* RNAs in sperm were compared between cases of successful and failed gestation following ART treatment. Data are shown as mean and standard deviation. *P*-values from unpaired Student's *t*-test. Abbreviations: FR: fertilization rate; CR, cleavage rate; ns, not significant.
